# The Significance of Enabling Human Consideration in Policymaking: How to Get the E-Ferry That You Want

**DOI:** 10.3389/fpsyg.2021.635722

**Published:** 2021-06-29

**Authors:** Alexander Berntsen, Simen Sæther, Jens Røyrvik, Mehmet Efe Biresselioglu, Muhittin Hakan Demir

**Affiliations:** ^1^Norwegian University for Science and Technology (NTNU) Social Research, Trondheim, Norway; ^2^Department of Sociology and Political Science, Norwegian University for Science and Technology (NTNU), Trondheim, Norway; ^3^Sustainable Energy Division, Izmir University of Economics, Izmir, Turkey; ^4^Department of Logistics Management, Izmir University of Economics, Izmir, Turkey

**Keywords:** e-ferry, technology neutrality, policymaking, electric mobility, transportation, sustainability, innovation, climate change

## Abstract

There is broad agreement in literature and policy that the transport sector needs to maximise electric mobility, in order to lower both energy consumption and greenhouse gas emissions. This ongoing transformation continues to require a high degree of technological innovation. Consequently, policymakers are striving to reward innovation in procurement tender contracts, in order to achieve sustainable innovation. At the same time, such contracts are often designed with a principle of technology neutrality in mind, to prevent any distortion of the market logic. This article suggests that it is misguided to try to perfect the logic of the tender system and that articulating contract that rewards innovation is no guarantee of a sustainable solution. Rather than being *technological*, the problem should be seen as *moral*: the mounting environmental challenge. Policymakers thus have clear ideas about the action needed based on what they, through moral conviction, consider to be appropriate action. This case study—conducted as a part of the EU H2020-funded ECHOES Project under Work Package 6—on the electrification of the Flakk–Rørvik ferry connexion reveals how policymakers were able to achieve the intended results: in this case, an e-ferry rather than a biodiesel ferry, in spite of, rather than because of, the tender system logic. They achieved this by involving stakeholders in the process with a continuous and uninterrupted dialogue. The project stakeholders were able to intervene in the tender system logic in favour of human considerations. We argue that this project was a success because human judgement, not system logic, was the driving force. By extension, we argue that systems will only allow policymakers to pursue moral issues to the degree that they allow human intervention.

## Introduction

In order to reach global decarbonisation objectives, it is important to include ferries and high-speed shuttles in the electric mobility revolution. Norway is recognised as a leader in this revolution, both with regard to electric cars and in the maritime sector. The charging of ferries, however, requires technical and infrastructural innovations. The battery packages are massive, and both the state-of-art smart energy technologies and charging infrastructure are involved in facilitating their charging. Flakk–Rørvik is considered to be one of the most difficult ferry connexions to electrify in Norway because of its weather and water conditions, as well as its intensive schedule.^1^ The decision-making process behind the electrification of this connexion is therefore of particular interest.

This article describes and discusses the successful implementation of electric ferries at Flakk–Rørvik,^2^ a 7.4-km ferry connexion. It is the seventh most trafficked ferry lane in Norway, located just outside Trondheim, connecting the city and mainland to Fosen at the other side of Trondheimsfjorden. This fjord has strong currents and temperatures at times reaching below minus 20°C. The new ferries need to be able to cope with these strong currents and potentially very cold temperatures. Because of this, these ferries require unusually high energy consumption compared with most others operating in Norway. The ferry lane was previously serviced by three liquefied natural gas (LNG) ferries managed by the ferry operator Fjord1 on a contract from 1 January 2011 to 31 December 2018.^3^

Fosen Namsos Sjø^4^ won the tender and began operating in early 2019, their contract lasting until December 2028. A shipyard on the Norwegian west coast delivered two hybrid ferries, both with a battery system of 2.2 MWh battery packs provided by Siemens.^5^ The charging infrastructure transfers energy equivalent to the capacity of one large Tesla per minute to the ferry batteries. This volume is a first-of-its-kind and requires intermediary dock-side battery banks. Both ferries are 103 m long, with a capacity of 399 passengers, 130 light-duty vehicles, and 10 trailers. They depart at 30-min intervals, and the journey lasts about 25 min. The inter-departure docking time is estimated at 6 min, allowing 5 min of efficient charging. Although the ferries are expected to be mainly powered by electricity, a hybrid solution was chosen due to the short docking time, strict schedule and challenging weather conditions. The ferries are commissioned to run on electricity at least 40% of the time, while the rest on certified biodiesel, and the operational rate is 43%. As a backup, a third reserve ferry is available, powered entirely by biodiesel. The operation is expected to be optimised during its first year by increasing the average electrical operating time.

Plans for a new tender for Flakk–Rørvik were initiated in 2015. The county^6^ authority was in charge of the process, with assistance from the Norwegian Innovative Procurement Programme. Together, they arranged dialogue meetings with interested parties, such as vendors and suppliers. The county owns the tender and opted for a contract tender that included eligibility requirements. Specifically, in order to be considered, proposals must adhere to strict emission limits. This eligibility requirement criterion enabled the county and AtB,^7^ their company responsible for the administration of transportation, to produce a standard price/quality tender, weighted at 70% price/30% quality, and without environmental requirements or specific technological demands.

One important result of the dialogue meetings was the understanding that a high degree of electrification of the ferry connexion is practically possible. Electrification is a central policy strategy in Norway, and the county could apply for funding from the state-owned support agency Enova toward the costs of establishing the electric infrastructure necessary for the dock-side charging. This enabled the county to guarantee the charging infrastructure for any proposals relying on electrification.

This article presents a real case of ferry electrification and explores the enabling process. E-ferries represent a great opportunity for potential economic savings and environmental conservation when compared with traditional diesel, diesel-electric and, to a lesser extent, LNG-powered ferries. In order to unleash this potential, however, policymakers need to find ways of facilitating the selection for e-ferries, as opposed to, e.g., biodiesel ferries; i.e., they seek strategies to *achieve the results they intend*. Here, they had to operate within the confines (and opportunities) of the tender system. In order to achieve their goal, it was imperative that stakeholders were involved through frequent dialogue, which increased the level of human judgement that was permitted and enabled them to influence the procurement process. The tender system logic itself does not inherently ensure sustainability, but rather encourages an emphasis on technological innovation, which may not necessarily result in greater sustainability when compared with technologically conservative solutions. Sustainability is thus a factor of *human interest*, not *systemic rule-following*, and this (or indeed, any) human interest is best expressed through clear personal communication, rather than attempting to mediate it through a system, although the reliance on systems is indeed unavoidable in policymaking. However, we argue that rather than attempting to encode policymaker intentions in these systems, policymakers should ensure that the systems are sufficiently open to permit interventions of human consideration.

## Materials and Methods

The research reported in this study was undertaken as a part of ECHOES' Work Package 6, coordinated by Mehmet Efe Biresselioglu, and Work Package 3, coordinated by Jens Olgard Dalseth Røyrvik—both are the authors of this study. This reported research also contributed to D6.3 and D3.1 of the ECHOES (2017, 2019) Project, in which Mehmet Efe Biresselioglu was also the lead author. Both authors, together with Muhittin Hakan Demir and Simen Rostad Sæther, contributed to these deliverables. More specifically, three authors of this article, namely Simen Rostad Sæther, Mehmet Efe Biresselioglu and Muhittin Hakan Demir, are also the writers of the “E-Ferries (Norway)” case study in D6.3 (ECHOES, 2019).

### Case Methodology

This article relies on an in-depth case study—a common method used in social science for the comprehensive examination of sustainable energy transition issues (e.g., Tiberio et al., 2020), chosen for its appropriateness in understanding the intentions, dependencies, and reflections, of the developments in this particular project. The development of a case description and case themes was supported by gathering and analysing in-depth and detailed data derived from interviews, documents and news media (Creswell, 2013). This way, results from case studies can be verifiable and representative (Geering, 2004).

The main source of data in this case study was semi-structured in-depth interviews with the stakeholders, presented in section Main Stakeholders, aimed to explore their perceptions and reflections. This method allowed us to uncover the process that led to this authentic case of electrification, and to explore the decision-makers' intentions and lessons learned.

The aim of this study was to understand the methods that enable policymakers to favour e-ferries over conventional alternatives. Representatives were given a central position when determining the sample. The choice of a qualitative method, and our selection of interviews, allowed us to enter into a dialogue with the key actors in the case, and thus enabled discussion of the intentions of those involved, and how they themselves assess their success and the strategies involved in achieving these results. Judgemental sampling was used, taking into account issues such as feasibility, time, and budget sensitivity. This involved selecting participants based on a specific criterion, i.e., direct involvement in the decision-making process, whether as top-level executive or mid-level manager. We, therefore, conducted semi-structured in-depth interviews with the County Project Manager, the Senior Adviser in State Support Agency and the Project Manager in the Innovative Procurement Programme.

The interviews followed a protocol design based on the literature review (ECHOES, 2017) and the existing policies analysis, which in turn followed the protocol below in line with the D6.3 of the ECHOES Project (ECHOES, 2019):

(a) The actual case to be described

(b) The existing alternatives to be analysed

(c) The approach used for the roadmap and solutions

(d) The different phases of implementation

(e) The results of the implementation

(f) The results from impact and diffusion

(g) Suggestions and recommendations.

Requirements for ethical and confidentiality from GDPR and the Norwegian legislation, including anonymity, were strictly complied with. All interviewees were provided with project information before signing a consent form. We conducted the interviews in Norwegian (the native language of the interviewees), and then anonymised transcripts were translated into English. The interviews lasted 60–90 min. They were all recorded, transcribed and coded in NVivo.

We produced a two-page debrief report after each interview, focussing on the emerging themes, the most central points, observations and reflections. These reports, transcriptions and translations were delivered to the wider project group. The debriefs were central for effectively implementing triangulation by analysing the interviews in the light of other data sources, such as policy documents and media texts from this case, and comparing the data with other data and initiatives. Triangulation methods ensured the robustness of our analysis.

### Background and Literature Review

The transport sector, one of the main consumers of fossil fuel, produces a substantial share of the EU's total greenhouse gas (GHG) emissions; it is one of the largest emission sectors, and it is the only sector that continues to *increase* its GHG emissions (EEA, 2016; Taefi et al., 2016). This sector is thus having an increasingly detrimental impact on the environment and climate change, as well as increasing its own dependence on fossil fuels (EEA, 2016).

In the EU H2020-funded ECHOES Project, we conducted a literature review focussed in large part on consumer behaviour and e-mobility, discussed at either the micro-, meso- or macro-level (ECHOES, 2017). Particularly relevant are findings at macroeconomic level, i.e., formal social units, which are themselves divided, for research purposes, into three levels: (1) formal social units that function as political decision-makers and/or energy suppliers; (2) collective decision-making units that are more formally structured, and which have relatively lower information and power symmetries; and (3) individual consumers.

The shift toward electric mobility seems to establish new market opportunities for those able to modernise infrastructure, digitalise technology and embrace innovation. As a consequence of changes in the manufacturing industry, service, and energy companies may also benefit from electrification, as these sectors will experience increased activity. In sum, innovation and development of new environmentally focussed technology in these sectors are predicted to create new employment opportunities (e.g., Haddadian et al., 2015; European Commission, 2016), which may be different from traditional ones (Røyrvik et al., 2015). Thus, electric mobility is the main focus of discussions concerning sustainable and energy-efficient means of transportation (Peters et al., 2011; Faria et al., 2014).

Our case study concerns the public transportation system, specifically e-ferries. An extended targeted literature search revealed a small number of relevant results,^8^ which largely agreed on the potential for electrification of waterborne transport, a sector with high amounts of energy consumption and carbon emissions. Of the very few case studies, none was as detailed as the current case. This lack of actual cases must be seen as a barrier to further adoption, as case studies confer significant leverage over policymakers.

The European Commission considers public transport a strategy for lowering emissions (European Commission, 2016), and therefore part of the solution to the European emissions problem, a position with which the IPCC agrees (Sims et al., 2014). In order to address the problem with emissions in the public transport sector, the goal set by the European Commission is emission-free urban passenger transportation by 2050 (i.e., no more conventionally fuelled cars in cities) and emission-free freight transportation in urban areas by 2030. Andong and Sajor (2017) conducted a case study on the Metro Manila, showing that urban sprawl and the associated workplace–home distancing in developing countries lead to greater public transportation use and consequently more emissions. They point out several interacting factors leading to increased carbon emissions from the transport sector. One of these factors is the low fuel efficiency of public transport. However, even with better fuel efficiency, emissions will inevitably rise with growing passenger volume.

Gagatsi et al. (2016) show that shipping, though technically fuel-efficient, is a major energy consumer and a significant source of carbon emissions due to the enormous volumes involved. Despite the relatively small number of ships compared with road vehicles, the energy consumption and carbon emissions of shipping are in fact not far behind those of road transport. Gagatsi et al. further show that maritime shipping represents 11% of the global transportation sector's petroleum use and highlights how this enormous volume is growing so fast that maritime carbon emissions are expected to surpass those of all land-based sources by 2030. If this is to be avoided, serious action is required.

Several of these arguments are repeated by Christodoulou and Cullinane (2020), calling attention to the potential for achieving large reductions of emissions through a combination of measures and policies, and stressing that no single measure achieves meaningful change on its own. The same problems form the basis of a case study of ferry lanes in Croatia by Ančić et al. (2020), who conclude that there is great potential for lowering these emissions.

Attitudes toward electric vehicles (EVs) are proven to have a significant impact on adaption rates, a topic that is integrated into the existing literature on collective decision-making units (e.g., Kaplan et al., 2016; Matthews et al., 2017; Biresselioglu et al., 2018a). Attitudes are found to be one of the key indicators, particularly vital to the decision-making process, and are considerably affected by technical aspects, among additional layers of factors and variables (Quak et al., 2016; Biresselioglu et al., 2018b; Usmani and and Rösler, 2015).

Charging infrastructure, considered to be of great importance for the adoption of EVs, is already one of the most investigated aspects in this field of research (e.g., Barlag, 2015; Laurischkat et al., 2016). Existing studies from similar contexts have focussed on the same topic (e.g., Caramizaru and Barlag, 2015; Laurischkat et al., 2016), underlining the strong need for knowledge about charging infrastructure as well as charging solutions. Thus, insufficient charging infrastructure is considered to be a core barrier (e.g., Barlag, 2015; Caramizaru and Barlag, 2015).

The characteristics of EV-usage represent vital information for decision-making (e.g., Norland and Ishaque, 2006; Ambrosino et al., 2015). Among the characteristics that affect the apparent potential to switch to EVs are vehicle use and tour patterns (Klauenberg et al., 2016).

There remain a number of operational challenges, for instance, grid issues for large fleets and limited availability of EVs. These represent important barriers (Quak et al., 2016). Companies working to transform their fleet to battery-driven EVs are similarly facing highly demanding major change processes (Laurischkat et al., 2016).

Among other significant factors needing to be considered, of course, are economic factors. Some, such as low fuel costs or efficiency of operation, are motivators, or push factors (Quak et al., 2016). On the other hand, other factors are barriers: high procurement costs of EVs; uncertainty about oil prices; energy prices; and the issue of limited, unreliable, and costly after-sales support (Laurischkat et al., 2016; Shao et al., 2016).

The role of operational and economic factors is magnified by issues pertaining to trust, in addition to the quality of, and accessibility to, information and knowledge (Quak et al., 2016; Biresselioglu et al., 2020). One final type, not to be underestimated, is environmental factors, such as environmental performance and vehicle noise level. For some segments, these factors may emerge as central to the decision-making process (Quak et al., 2016).

### Policy Analysis

We have established that the transport sector is a significant source of CO_2_ emissions in the EU, and an area highly prioritised in the idea of a coming transition to a low-carbon/low-emission society. In the Strategic Energy Technology (SET) plan (European Commission, 2014), decarbonisation in the transport sector from fossil-fuelled to electric mobility has evolved into a highly prioritised research and innovation area. Electric mobility comprises a large and diverse domain of expertise: from plug-in battery electric cars and plug-in hybrid electric vehicles, to electric bicycles and motorbikes, electric busses and transporters, and electric aviation and ferries. This diversity is so far not recognised in most current policy documents, which focus on electric or hybrid passenger cars.

Various aspects of the electrification of the transport process are prioritised in a number of EU-level initiatives, such as the European Economic Recovery Plan (European Commission, 2008) and the Green Car Initiative (European Commission, 2012). According to the European Commission, in policies supporting the transition to battery-powered vehicles, the overall solutions revolve around technological optimisation and market development.

One future obstacle or challenge of the transition will be establishing and maintaining a sufficient charging infrastructure and plug-in solutions, and another is the need for improving battery reliability and durability. Among other numerous additional related topics that require attention are supercapacitors, reducing battery weight and volume, safety, and cost reduction (European Commission, 2021).

With the H2020-funded E-Ferry Project (E-ferry, 2015), the EU funded €15M of the €21.3M total cost. This project created an e-ferry connexion between Ærø and mainland Denmark. The e-ferry operating cost is estimated at between 24 and 36% lower than that of a diesel or diesel-electric ferry. Additionally, e-ferry battery technology costs are decreasing, and the availability of charging systems and grid infrastructure is inevitably increasing, pointing to even greater future savings. The e-ferry resulted in significantly reduced pollution compared with a diesel or diesel-electric ferry. Finally, the project reports high passenger satisfaction and thus produces social as well as financial and environmental benefits.

## Case Study

The Flakk–Rørvik e-ferry is a case of policymakers successfully achieving their intended results, i.e., the implementation of new technology and environmental policy, within the confines of the tender system. The main data for this case study are the interviews with stakeholders described above, namely the County Project Manager, the Senior Adviser of the State Support Agency and the Project Manager in the Innovative Procurement Programme.

On 5 February 2016, AtB registered the ferry contract tender in the Doffin system, the formal Norwegian Database for all public procurement. In April of the same year, the first tender conference was arranged, and the deadline for proposals was 25 April 2016. The assessment process took place during weeks 17–23 of 2016, and the contract was awarded at the end of this period. After a 3-week period for appeals, the contract was finally signed on week 26, 2016.

### StakeHolders

**Figure 1:**
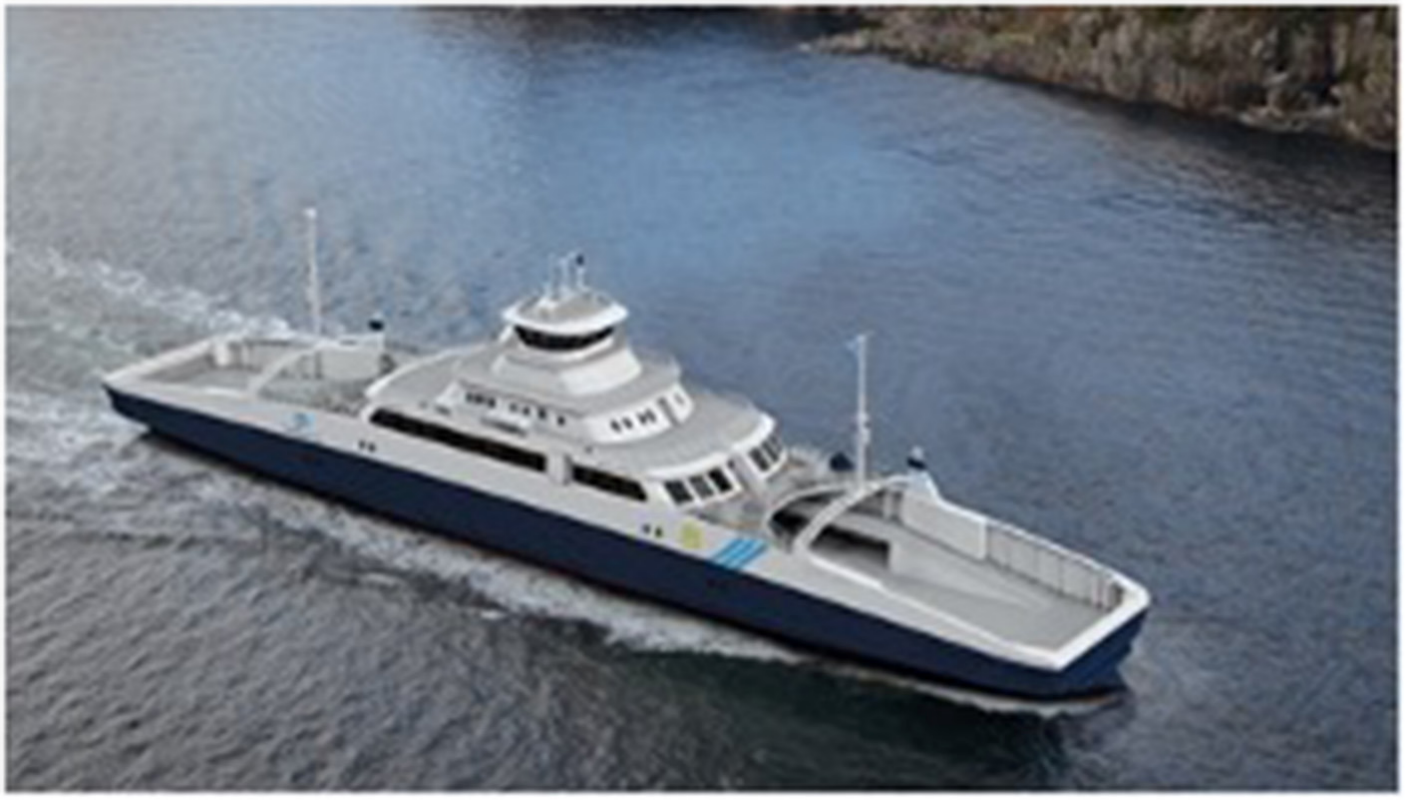
*E-ferry*. Image: Fosen Namsos Sjø.

**Figure 2:**
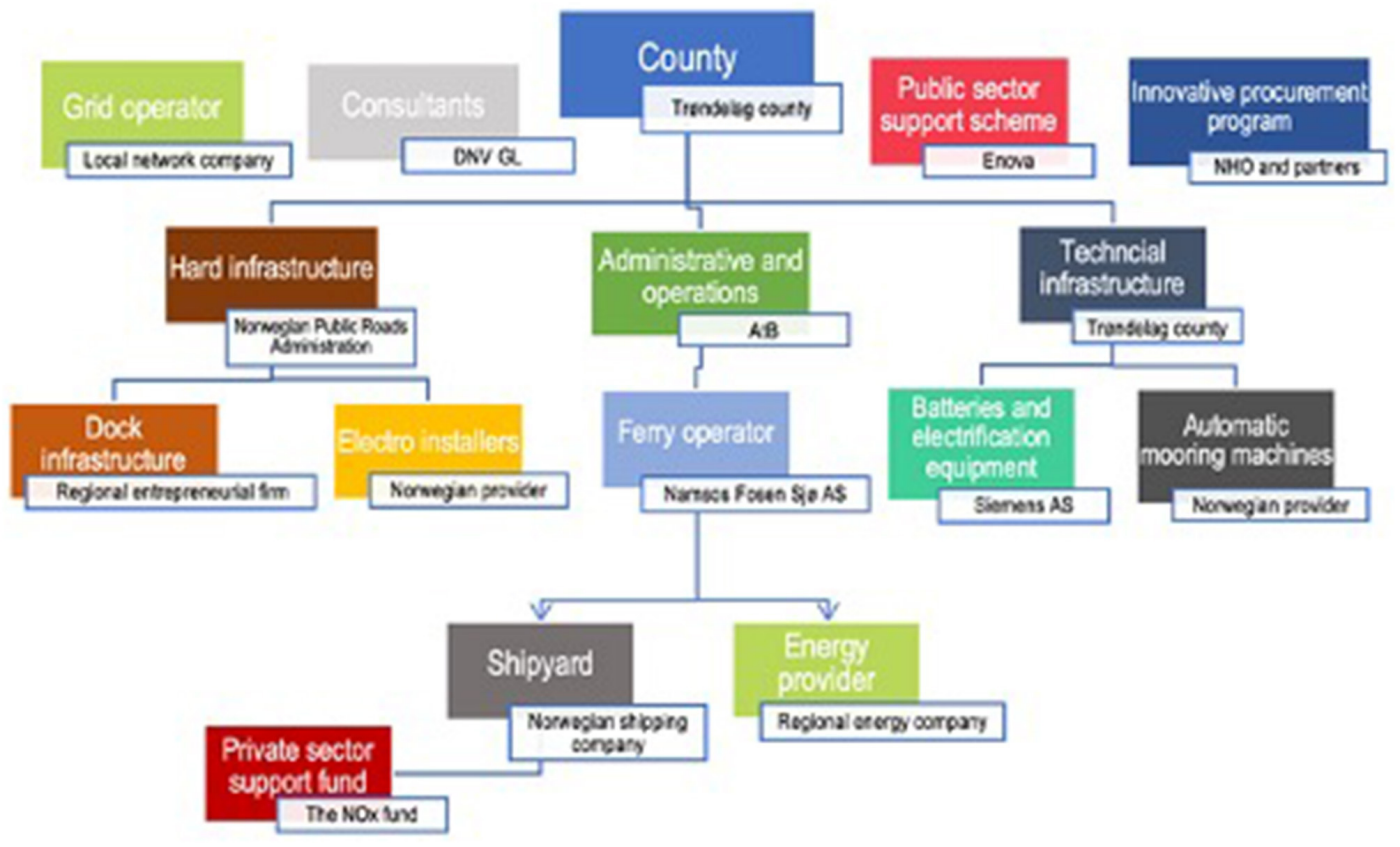
*Stakeholder map*. Visual representation of the relevant stakeholders in the Flakk–Rørvik project.

This case study shows the realisation of the e-ferry Project, in which stakeholders successfully reached the desired solution, despite a system that only takes into account specifications, rather than the concrete wants of stakeholders. See Figure 1 for an actual image of the e-ferry discussed in the case. The success was due to the ability of stakeholders to mediate these wants. It furthermore shows how this success depended on the stakeholders' different abilities to promote their interests through dialogue with each other. Figure 2 shows a map of stakeholders for the Flakk–Rørvik Project derived from the interviews.

#### Main StakeHolders

**Trøndelag County**, the home of the Flakk–Rørvik ferry connexion, is a key stakeholder in this case and employs the two project managers. In Norway, ferry operations connected to county roads are managed by the counties, while those connected to national roads are managed by the Norwegian Public Roads Administration. In our case, the county owns all resources, including the road-dock infrastructure, the publicly announced operations tender, and 100% of the stocks in AtB, the transport company that manages Trøndelag's public transport. The county and AtB furthermore hire consultant firms, such as DNV GL, to perform the complex calculations necessary for meeting the energy consumption and grid capacity requirements for new ferries. The project's level of novelty demands a high level of management and coordination of the solutions and actors, leading to the unusual situation of both project managers being involved from the office and in the field.

**The administrative company, AtB**, is responsible for managing the public transport service through planning, purchasing and marketing. Their operations include running the county buses (including school buses), trams and boats (including ferries), among others. AtB is registered as a limited company, entirely owned by Trøndelag county (AtB Website, 2018).

**The ferry operator, Fosen Namsos Sjø AS**, won the public tender for operating the Flakk–Rørvik ferry route with their two newly acquired hybrid ferries from 1 January 2019, with a backup ferry running on biodiesel. The hybrid ferries were purchased from a local shipping company, *Myklebust Verft*, built in one of their shipyards in Western Norway. Myklebust Verft received financial support from the NO_x_ fund, a funding scheme for the private sector, administered and financed by the sector itself. Norway has for some time been investigating the possibility of establishing a similarly structured fund aimed at CO_2_ emissions. The ferry operator purchased the required electricity from a local provider. The project managers stated that this electricity was cheaper than the biodiesel required for the generators, providing a strong incentive to maximise the rate of electrification.

**Enova SF** is owned by the Norwegian Ministry of Climate and Environment and is the Norwegian government's main tool for providing economic incentives for reducing GHG emissions and supporting the development of more efficient energy technologies, as well as for ensuring the reliability of the energy supply.

The county is eligible for Enova funding toward the dock-side infrastructure costs. There are, however, several criteria for Enova funding, with regard to eligibility, the extent of funding and the potential to innovate or achieve otherwise unattainable results (Enova Website, 2018).

**The National Programme for Supplier Development**, often referred to as “the Innovation Procurement Programme,” was set up to accelerate the rate of innovation and development of new solutions through strategic public procurement and, at the same time, contribute to opening up new markets for these innovations (Innovative Procurement Programme Website, 2018). The programme is a collaboration between important public and private sector entities, aiming to harness their unique strengths, networks and goals.

**The Agency for Public Management and eGovernment** (Difi) is involved in the field specifically to support the development of tools and guidance on public procurement, particularly with regard to innovative public procurement.

**The Norwegian Association of Local and Regional Authorities** (KS) links projects to local and regional authorities in order to stimulate actors toward innovation in public procurements.

**The Confederation of Norwegian Enterprise** (NHO) links projects to actors in the private sector. NHO host the Programme Secretariat, giving direct access to relevant suppliers from specific sectors.

**Innovation Norway** (IN) is the Norwegian Government's tool for enabling innovation and development of enterprise and industry. They support companies in developing their competitive advantage and enhancing innovation.

**The Research Council of Norway** (FR) is the government authorities' chief advisory body concerning research policy issues. They annually distribute approximately nine billion NOK for short-term research andinnovation activities.

In this case, the programme was initiated in advance, thereby connecting and engaging actors and facilitating their dialogue with Trøndelag county and AtB in the early stages of the procurement process.

#### Other StakeHolders

Several other business actors and stakeholders are present on the hard dock-side infrastructure. Management was outsourced to the Norwegian Public Roads Administration, who was tasked with handling tenders and contracts with subcontractors for the dock-side infrastructure and electro-installers. The contract to build and upgrade the dock-side infrastructure was awarded to a regional entrepreneurial firm.

The county managed the technical infrastructure itself. Tenders were announced for the dock-side land-battery system and the automatic mooring machines. The only option for providing sufficient charging for the docked ferries was to supply high-voltage power from a land-based battery buffer. Siemens supplied the battery systemsto the ferry operator.

### Planning and Development: Interests and Technology Neutrality

Procurement processes are designed to hedge against competition-distorting factors through various strategies, e.g., a principle of technology neutrality—viz. tenders cannot openly favour more-or-less-specific technologies over other technologies that are equally effective. At the same time, it is obvious that policymakers favour certain alternatives over others both in terms of technologies and in broader terms concerning solutions. This may be because certain solutions are understood as inherently superior or as instruments—i.e., accepted as a way of achieving a goal. An e-ferry is a clear case of the latter, being perceived as an instrument toward a goal of great moral import: the environmental and climate goals of society.

The Flakk–Rørvik ferry lane is in a county with its own unique approach to how transport is organised. Some counties use technology-specific orders in their tenders—i.e., “only electric need apply”—while others try to maintain a principle of technology neutrality. The process in this case, however, was described neither as top-down nor as bottom-up, but as having dynamics resembling a wave.

“It was by no means an order that we received, certainly not in this county. It will vary a lot from county to county. You don't have to go further than to our closest county, where it was much more of an order. So there the county politicians went like, we are going to have electric ferries, so fix it, that sort of thing. […] Here it has been more from the transport department and the county administrative director to the politicians and down again, so it's been more like waves. […] It has been a lot of sparring, much back and forth between different actors. So there is nothing clearly defined, neither top-down nor bottom-up that I can see at least. And it has not come from the ferry crew either if you take it all the way there.”

**Interview Norway, County Project Manager**

The manager argued that the reason for this dynamic was due to the overarching goals of the project:

“It has nothing to do with transport directly, it was about greenhouse gas emissions and stuff like that that really led to this. It never stood anywhere that we should have electrical ferries.”

**Interview Norway, County Project Manager**

When asked to expand on this, the project manager focussed on the tender's eligibility requirements and pointed out that the biodiesel option would have been selected had it turned out to be the cheaper solution:

“One should strive to be as technology neutral as possible in the tenders for the ferries. So, if, let's say it, and then we would have been disappointed for sure, but if the ferry offer with biodiesel was the cheapest, it would have won. Because in order to be eligible you had to fulfil the requirements right, but it wasn't the cheapest, so…!”

**Interview Norway, County Project Manager**

In Norway, it is now mandatory to include low-emission technologies in all new ferry tenders, although this ruling includes no provision for funding. To alleviate this situation, the state-owned support agency, Enova, was made responsible for providing additional funding for such projects. The promise of funding was designed to have a triggering effect, and Enova is furthermore responsible for providing expertise in mapping project needs and feasibility—in collaboration with other consultants involved. The senior advisor at Enova explained that there are many factors to consider in conducting a successful tender.

Enova is therefore generally a central part of the tender process, having developed extensive in-house capability based on their experience of following and facilitating a number of tenders. After the preliminary reviews and calculations, the county applies for funding from Enova in cases in which the project's innovation potential is the key factor. If granted, up to 40% of the infrastructure costs are funded by Enova, with the justification that:

“Our goal is, on the overarching level, we're heading for a low-emission society, and then we have to change the market in order for us to get there. Thus, we have market transformation goals as guidelines for our work throughout the organisation, and for maritime transport, this is zero emissions, competitive zero emission vessels.”

**Interview Norway, Senior Adviser in State Support Agency**

Enova is regulated so as to prevent the distortion of competition, but they are permitted to support a project that would be otherwise unfeasible, and continue support until the project is independently profitable. The senior advisor explained that their goal is to ensure that the supported projects are competitive and to support technologies that are considered financially sustainable, i.e., projects they predict will eventually be profitable. In this regard, another rationale was also considered important in their evaluation:

“When we started out, our rationale was to support these projects because we wanted to build the entire value chain of battery technology. We have great potential to do this in Norway. The value added from the fact that the entire value chain is located here is huge. […] Here we really have the opportunity to create jobs and create added value and technology development in Norway. And when we control the whole value chain, it's also easier to create market change. […] When we support ferry connexions, it's not only in order to have as many electrified ferry connexions as possible but so that we can eventually include the entire maritime sector.”

**Interview Norway, Senior Adviser in State Support Agency**

Another central actor, the national programme for supplier development, was also an early collaborator on the Flakk–Rørvik Project. The project manager stated an interest in ensuring and facilitating a professional process for public procurement. The role of this programme is to be located between public and private/business actors, ensuring mutually innovative solutions and beneficial partnerships.

The supply chain for ferries in Norway is relatively small. The number of actors is manageable for the involved parties, underlined by both the project manager of the national programme and the senior advisor from Enova. Furthermore, they both drew attention to the specific, and special, ways that these actors try to maintain a balance of cooperation and competition:

“Very manageable [number of actors]. And the industry [actors] know each other very well. And they often have open communication lines among themselves. There is, of course, a little competition, I mean they compete, but at the same time, they cooperate a lot. They are very open about that, especially on the development side, I think.”

**Interview Norway, Project Manager in the Innovative Procurement Programme**

“What is special about Norway is that we have clusters working together, essentially cooperating when they can and competing when they have to. There's something Norwegian about this. And many have stressed that this success could not have happened in any other place than Norway, because they simply don't have that [business] culture.”

**Interview Norway, Senior Adviser in State Support Agency**

At a very early stage in the process, the county, together with the Innovative Procurement Programme, invited the stakeholders to dialogue meetings. Respondents were unanimous that this step was considered pivotal for the success of the project. The county project manager elaborated:

“The county conducted [the market dialogue] through dialogue conferences and one-to-one meetings, and that is a very open process that is two parts, where we go out and present openly what we are searching for, and we also let the market actors into closed cubes where they can speak. So in those, we will not take any reference or notes, so there they are at liberty to speak, and what comes out there is between those who are there. So then, we go out of there and make an assessment based on the information we have gathered there. So we get a lot of different actors' perspectives and get a lot of input.”

**Interview Norway, County Project Manager**

The project manager of the Innovative Procurement Programme underlines the importance of careful considerations at this stage of the project. There are a number of challenging decisions, and different options ought to be examined, thereby highlighting the need for, and relevance of, a programme focussed on process knowledge. The programme's manager also mentions the importance of frequent dialogue, in ensuring a dynamic and flexible process:

“We often assist in writing a so-called dialogue-note that we are working out when we invite actors to dialogue. Once we have come this far then, we may have to work a lot with the need. Then we provide input on how they can do it, how can they explore the need, how can they clarify the need, etc. Then there is the dialogue, yes. So, there we have the dialogue-note, we can help you prepare and either write a little on it or provide feedback on it, as well as to create a good program for a dialogue conference and subsequent dialogue, so how can we facilitate that in a good way. And here there are many ways to choose from. One can have the providers come in very early, also perhaps in the need phase sometimes, in order to get the common understanding of the need. Or they can be drawn into the dialogue phase. Also, we are a discussion partner along the way in the dialogue phases. Perhaps especially important after they have gone through the dialogue phase when they are sitting there with a lot of info and impressions and are thinking; Wow! What do we do next? So, we talk with them then and after that, we can talk about, for instance, what can be done in the new contracts? […] So, there are a lot of questions here.”

**Interview Norway, Project Manager in the Innovative Procurement Programme**

The county manager simply states that in a development phase, inevitable hindrances and difficulties will need to be overcome. He further explains that even small and seemingly insignificant infrastructural obstructions may have complex consequences:

“So, well, you have, all these simple hurdles with the infrastructure to be able to dock these ferries, then you have to supply power. You actually have to sign a supplier that is willing to deliver whatever equipment you need and manage it, and you need some development in the market. And you need acceptance on all levels. Then you need a ship-owner that is willing to deliver an electrical ferry. You then need to get an operator or administrative company that is willing to bet on it, for their tender. You need politicians who are willing to approve, and you need support schemes that can help you fund it by allocating some money for it. So, there are plenty of things that are not straight forward, for sure.”

**Interview Norway, County Project Manager**

The data analysis from the project interviews identified three main success criteria: one was the early market dialogue described previously; another, the county council's very ambitious climate goal—halving GHG emissions by 2020—in a context where transport accounts for close to 85% of emissions. This caused the county managers to focus intensely on each segment. The final success criterion identified for the Flakk–Rørvik Project was the tender's incorporation of a climate bonus arrangement.

As a path to success, Enova's senior advisor highlighted the impact of active dialogue between the county council and the city's technology actors, combined with the ability to act on this dialogue. The rationale for the climate bonus model was clarified by the county project manager:

“We had that absolute requirement and the fact that we decided that we should not have any environmental criteria in the tender, because that is our assessment, and well yes it becomes problematic. Because you see that when you set that absolute requirement that is an eligibility requirement to enter the tender, and you make that, you have no incentive to go further, right. So, we added that climate bonus there as a carrot instead of a stick.”

**Interview Norway, County Project Manager**

The county manager does not suggest this bonus arrangement as being intended as a formal challenge to the bidders. Rather, they explain and underline the spontaneity of the decision, as a spur-of-the-moment idea. Both ship owners and operators responded positively, and the arrangement was therefore immediately added to the contract. According to the manager, this also had a positive outcome for the ship owners, whose costs would be lowered by switching to electricity. They would get extra compensation, in effect, be paid extra to save money. However, compared to the NOK 30 million saving with the NOK 2 billion contract, as the manager points out, the sum is relatively modest (€1 ≈ kr10.5 at the time of writing).

The tender's climate bonus arrangement was specifically hailed by the project manager of the Innovative Procurement Programme as a very significant innovation. The flexibility of the contract allowed for continuous improvements, including upgraded batteries and optimising the rate of electric operation:

“What was special about this particular procurement, is that in the contract it says that during the contract period the supplier-vendor can make sure to include ferries that emit less and less continually. So, it's not a static solution that they've gone for. So, they managed to create a contract that allows them to include continual technological development with respect to emissions. And that was a great innovation really in this context.”

**Interview Norway, Project Manager in the Innovative Procurement Programme**

They also underlined the need for flexibility to succeed throughout the whole system.

“The support instruments are a bottleneck now. Because we don't really have tools suitable for innovation processes. We have instruments that are suitable for technology development, but not innovation. This becomes clearer and clearer for me, every day. The Research Council is not suitable for innovation. It is suitable for research! […] Research is desperately important, but we are not done with innovation at that stage […] There have to be flexible means that need to be available when we (the innovator) need them. So we cannot have an application that you apply for funds, so you have to spend a lot of resources on, and it takes a year before you get it. Then it's too late. When the air goes out of the balloon, it's gone.”

**Interview Norway, Project Manager in the Innovative Procurement Programme**

We have so far established that in the planning and development phase, the procurement process was designed to promote the idea of technological neutrality, intended to counteract the distortion of competition through rival stakeholder interests. However, quite apart from the technical aspects, the e-ferry might also be regarded as an instrument for a moral end: in this case, an end that the county was able to achieve by out-manoeuvring a supposedly neutral and objective tender system logic. The empirical evidence suggests that this was made possible through a process involving dialogue, cooperation and an overall notion of the project's significance. This achievement was only possible because the system was open and flexible, and permitted human interventions and considerations to overturn the system logic.

### Implementation and impact

The actors involved conclude that the e-ferry emerged as the winner because of the dialogue and collaboration that characterised the process. Additionally, the stakeholders feel a sense of pride and ownership, precisely because the technology is perceived not as neutral, but rather as the most appropriate solution. The success of the implementation was founded upon the moral conviction that this project is *the morally acceptable thing to do*.

There were also obstacles in the implementation phase, and the most serious of these was the dock-side infrastructure. The charging system was still incomplete when the two ferries started operating in January 2019 and was eventually finished in mid-June, which means that the ferries relied on diesel alone for the first 6 months. Nevertheless, AtB's guarantee that 43% energy consumption should be electric^9^ still applied across the whole period of the contract.

Interestingly, the project manager focused on the informal aspects as key to the success of the process, firstly on their feelings of being involved:

“Actually, what goes on is ownership and pride. We see that now, that is it incredibly important. You notice it when someone who doesn't quite have the ownership and then gets a little. And if someone has ownership, then it will be good. So you want to create a showcase window (for your solution, product). All the actors that are part of this ferry project get that showcase window. So they can point to that and say, we or I made that part or system right there. So now, you have many that are very proud of what the work execution they have done here and contributed to. So that is really important.”

**Interview Norway, County Project Manager**

Furthermore, it seems that the group shared a common goal, crucial for the direction of the process:

“It is all about making sure you have a common goal to succeed, so the actors are united in saying that we will solve things that come up. Moreover, that we manage to distinguish what is contractual and legal and problematic from what is possible to solve here and now, so you just do it, note it, and sort it out afterward. […]”

**Interview Norway, County Project Manager**

He further points out that the biggest challenges the project faced were related to difficulties with communication:

“I guess one of the biggest challenges for the project has been that sometimes someone is waiting on someone else that needs to do something, but not telling us that they are waiting.”

**Interview Norway, County Project Manager**

The main parts of the case study were conducted during the latter part of 2018 before the project was implemented in January 2019; therefore, it was difficult to assess the success of the project. The county and the ferry operations considered that at least a year should pass before firm conclusions could be drawn. In this context, it is nevertheless the case that the decision to choose e-ferries in itself can be considered a success, and according to the project manager, it was already clear in 2018 that the project was a success in terms of the communication of ambitious climate and environmental objectives by actors. This attitude was evident not only among the managers but also among employees in the various organisations. This gave decision-makers hopes for similar ambitions extending across the whole transport sector, as well as other routes and ferry operators. Some of these transport sectors—such as speedboats—have other, and sometimes more challenging technical and organisational issues, and it is, therefore, crucial to have ambitious targets to aim for.

The most obvious of the impacts of the project was on ferry connexions in the areas in and around Trøndelag, summed up in the argument: “if we were able to achieve this very ambitious project, electrification of the other ferry connexions should be relatively simple.” By taking on the most challenging connexion first, the county effectively initiated a solution to the problem of electrifying ferries. Siemens decided to locate their new state-of-the-art battery factory in Trondheim in no small part due to their involvement with the electrification equipment and battery systems for the Flakk–Rørvik connexion.

“When that first county contract was signed, I think a lot of heads turned and asked, if they dared to sign such an agreement, they wonder if they could achieve it too. Moreover, it is clear that this ferry lane is really demanding, so if we succeed here, we will succeed on all our other ferry lanes in the county. […] There are a lot of ripple effects, the new battery factory that is established in Trondheim, it has our ferry lane as a showcase window, if you are going to buy an electrical ferry with Siemens technology, you will be invited to come and see it here. So, there are a lot of those types of things that many are not aware of.”

**Interview Norway, County Project Manager**

The project manager of the Innovative Procurement Programme observed several ripple effects and in-house experiences. This project is highlighted as an example, which shows the potential of well-thought-out projects in achieving challenging goals. The senior advisor in the state-owned support scheme agency related that the biggest in-house ripple effect was a change of orientation from simply measuring energy consumption reduction in absolute numbers to a complete change in market conditions.

On a final note, the success of this case has undoubtedly inspired some of the more recent developments in Norwegian ferry connexions. The Norwegian Public Roads Administration awarded a developmental contract for the first hydrogen-electric ferry to the ferry operator Nordled (the operator of MF Ampere, the world's first fully electric ferry), on the Hjelmeland–Nesvik–Skipsavik connexion in Rogaland county, in south-western Norway. From 2021, the ferry is expected to use equal amounts of energy from hydrogen and from batteries charged from the dock. Additionally, the government has decreed (Regjeringen, 2020) that one of the longest Norwegian ferry connexions, Bodø–Moskenes, must employ hydrogen ferries from 2024.

The implementation appears as having favourable outcomes both for the Flakk–Rørvik ferry lane itself and for the general situation regarding ferries. In this case, the stakeholders were thereby proven right in their conviction. The solution must be seen as the most appropriate option precisely because it was founded on human judgement.

## Discussion and Concluding Remarks

### Technology Neutrality and Innovation: for Better and Worse

The tender system and economic discourse in Norway tend to favour technology neutrality, and this was a specific requirement in Trøndelag County. This causes a dilemma, in that while it may help move the focus from technology to sustainability, it may also make it more difficult to achieve set goals. We saw that interviewees worried that the tender logic, if unguided and uninfluenced, might have favoured a diesel ferry. Fortunately, in this case, the intended goals were realised, but this was in spite of rather than because of the system logic.

The system emphasises the importance of innovation in proposed solutions. This too produces a dilemma, in that innovation is good only if it allows goals to be achieved. If the system blindly rewards innovation, it risks promoting unsustainable solutions on account of their innovative capabilities. Innovation is not synonymous with sustainability, and treating it so can be dangerously misleading in cases of such paramount importance to the sustainability of human life. We saw that Enova rewards innovation, which they emphasise as intrinsically beneficial; however, equally, we saw innovation criteria supporting policymakers' aims, i.e., tenders requiring low-emission solutions.

To ensure that policymakers succeed, flexibility is important, and complaints throughout the project typically revolved around the perceived lack of flexibility in the process. The interviewees stressed the importance of pride and ownership, reflecting people's need to feel involved in the project. These issues are interconnected and culminate in the desire to exercise human considerations in projects of great societal importance. This ability to exercise judgement is, in turn, necessary in order to curb the role of technology neutrality and innovation, allowing the balance to be tipped in favour of policymakers.

### A Case of Success

An overwhelming number of studies demonstrate that investment in the construction of e-ferries rather than conventional diesel ferries will result in massive reductions in energy consumption and GHG emissions from the maritime transport sector. Said reductions will be of an exceptionally important magnitude if Norwegian national policy were to favour e-ferries, opening the door for potential worldwide replication. At the same time, it is clear that greater further efforts are needed to ensure the continued electrification of the maritime transport sector. This article shows some of the important aspects concerning (a) stakeholders that inevitably take part in this process, (b) the procurement principles at play and (c) the actual implementation of such a project.

On 26 November, The Norwegian Broadcasting Corporation (Norsk rikskringkasting AS) reported^10^ that the Norwegian government has announced that, from 2023, all new ferry tenders will require low- or zero-emission ferries. Erna Solberg, the Norwegian prime minister, highlighted the reduction of Norwegian emissions as the primary motivation for this new demand, and also allocated an undisclosed amount of public funding to this end.

### A Case for Human Judgement and Flexibility

This case study is primarily a study of the success of policymakers achieving the results they intended. This success was, however, not guaranteed or safeguarded by the system logic. It is imperative that the success of the case is neither considered as a success of the system with which the project participants had to contend, nor considered as an argument in favour of technology neutrality or innovation as intrinsically valuable in and of themselves. By gaining an insight from decision-makers and public officials, we are able to understand their intentions and the perceived effects that e-ferries might have beyond this one specific case. The case can also serve as an inspiration for policymakers and decision-makers in general, and also other sectors facing the challenge of sustainable transition.

While it may superficially seem that the technology and policymaking work together in tandem toward intended results, in reality, it is more complex. Technology works to maximise technological progress and innovation, and policymaking seeks to leverage this by rewarding innovative technologies, e.g., the “climate bonus” reference in the case, or how Enova rewards innovative technologies that help society transition to low-emission solutions. *Prima facie*, it appears that technologies use policymaking to advance technology and that policymakers use technology to advance policymaking—and to some extent, this is indeed true. What lies at the core of the desire for an e-ferry on the Flakk–Rørvik line cannot, however, be explained as merely technological; it is a *moral* point: we need to transition to a low-emission society in order to allow the planet, including, of course, its people, to survive and flourish. This moral point must then be *techno*-*logically* mediated through the tender system. The result is the amoral specification for a technological solution. The policymakers, however, aim not at a technological solution, but a moral solution. In the policymakers' intentions, the e-ferry as technology is an arbitrary element in the moral problem of humans attuning themselves sustainably to their environment. However, in the technological system, this relationship is inverted, so that the moral problem becomes the arbitrary element, and the technology becomes primary. We contend here that policymakers regard e-ferries as the means to an end, i.e., environmentally orientated travel, whereas, from a technological point of view, the environment itself becomes the means of triggering financial rewards through mechanisms such as innovation funding and climate bonuses.

Policymakers should be *informed*, not *formed* by science. Science can never legitimise moral decisions, but it may inform and thereby assist us. Correspondingly, scientists should not have their activity shaped by policymakers. The legitimisation of political strategy cannot be the scientists' creed. It is, hopefully, not controversial to assert that science should not be subordinated by immoral objectives; a society's moral convictions always precede both science and policymaking. The role of policymakers here is to reflect the morals of the society. Since, in the case in point here, much is at stake, regarding present-day environmental challenges, it is important that policymakers are as well informed as possible, which is the role of science today. In order for this to be successful, the importance of dialogue cannot be overstated, as is reflected by the case study interviewees. Dialogue is, as Feyerabend (1987) argues, essential to science in a free society.

In the end, human interests can only be articulated and preserved by exercising the human quality of judgement that stems from the—human—capacity for care. Disembodied machines and systems operate by following rules expressed in the propositional knowledge they receive as input, but while a well-designed system will indeed identify the correct solution to a well-formed problem, its greatest weakness from a human point of view is that the system simply has no investment in the outcome. Thus, such systems are in constant need of human intervention if they are to provide the desired outcomes. To preserve human interests, systems must have the flexibility to allow for intervention, thereby maximising human considerations. To do this is to facilitate dialogue, which requires ensuring that all parties are fully involved. This relationship is reciprocal; this system flexibility is necessary to ensure the capacity for dialogue and, conversely, dialogue, to ensure judgement.

## Data Availability Statement

The datasets presented in this study can be found in online repositories. The names of the repository/repositories and accession number(s) can be found at: https://www.echoes-project.eu.

## Ethics Statement

The studies involving human participants were reviewed and approved by it has undergone the Ethics Committee of the H2020 project ECHOES, and the Norwegian committee NSD. The patients/participants provided their written informed consent to participate in this study.

## Author's Note

The research reported in this study was undertaken as a part of ECHOES' Work Package 6, coordinated by MB and Work Package 3 coordinated by JR, both are the authors of this study. This reported research also contributed to D6.3 and D3.1 of the ECHOES (2017, 2019) project in which MB was the lead author as well. Both authors together with MD and SS contributed to these deliverables. More specifically, three authors of this article, namely, SS, MB, and MD are also the writers of the E-Ferries (Norway) case study in D6.3 (ECHOES, 2019).

## Author Contributions

All authors listed have made a substantial, direct and intellectual contribution to the work, and approved it for publication.

## Conflict of Interest

The authors declare that the research was conducted in the absence of any commercial or financial relationships that could be construed as a potential conflict of interest.

## Footnotes

^1^About the connection weather and currents: NRK Website “Her er fjordens svar på tesla.” Available online at: https://www.nrk.no/trondelag/bygger-hypermoderne-ferjeleier-for-landets-kraftigste-elferjer-1.13884818 (accessed April 2, 2021).

^2^Formal and technical aspects about this ferry connection can be read on the basis for competition for operating the connection: AtB Website Basis for Competition (2019). Available online at: www.mercell.com%2Fm%2Ffile%2FGetFile.ashx (accessed April 2, 2021).

^3^About the new ferries: https://www.tu.no/artikler/kleven-bygger-hybrid-ferger/364498.

^4^About the company: Fosen Namsos Website “About us.” Available online at: http://www.fosennamsos.no/om-oss/category163.html (accessed April 2, 2021).

^5^The technical details (only in Norwegian) can be downloaded: Fosen Namsos Website ≪Munken er Her≫. Available online at: http://www.fosennamsos.no/aktuelt/mf-munken-har-ankommet-norge-article9720-892.html (accessed April 2, 2021).

^6^The county about the ferry connection: Trøndelag Fylke Website “Ferjelading.” Available online at: https://www.trondelagfylke.no/nyhetsarkiv/forst-i-verden-med-hoyspent-ferjelading/ (accessed April 2, 2021).

^7^“AtB” is the legal name of the company; it is an expression meaning “getting from A to B.”

^8^Web of Science only yields four hits on “e-ferry.” There were vastly more hits on broader search topics. This includes 330 hits on “ferry” and “environment,” 153 hits on “ferry” and “policy,” 871 on “public transport” and “electric,” and 90 on “e-mobility” and “transport.”

^9^https://www.atb.no/ferje/, visited 2020-11-24.

^10^https://www.nrk.no/norge/stiller-krav-om-lav–eller-nullutslippsferger-innen-2023-1.15261914.
